# Lactylation in influenza a virus infection: Current evidence, knowledge gaps, and future perspectives

**DOI:** 10.1080/21505594.2026.2712045

**Published:** 2026-08-02

**Authors:** Yu-Mei Chen Yan, Zhijun Li, Haiyan He, Moding Wu, Haolin Yang, Fanhua Wei

**Affiliations:** a School of Animal Science and Technolog, Ningxia University, Yinchuan, China; b Key Laboratory of Animal Disease Prevention and Control & Veterinary Biosafety of Ningxia, Yinchuan, China

**Keywords:** Influenza A virus, lactylation, SIRT1, metabolic reprogramming, immune evasion, antiviral therapy

## Abstract

Influenza A virus (IAV) is a major respiratory pathogen causing seasonal epidemics and pandemics, posing serious threats to public health and livestock. The high mutation rate of IAV leads to vaccine mismatches and drug-resistant variants, underscoring the need for novel antiviral strategies. This review examines the role of lactylation in IAV-host interactions, focusing on three core questions: how IAV induces lactylation, how lactylation reshapes antiviral immunity, and how IAV exploits lactylation for immune evasion. Key findings include lactylation of viral vRNP components required for efficient replication and the host deacetylase SIRT1, which suppresses IAV replication by removing these lactyl groups. IAV counteracts this defense by downregulating SIRT1 expression. Lactylation also regulates cGAS-STING, RLR-MAVS, and IFN signaling pathways. The therapeutic potential of targeting lactate metabolism and SIRT1 is discussed. Understanding lactylation in IAV infection may open new avenues for antiviral drug development.

## Introduction

IAV is an enveloped, segmented, single-stranded negative-sense RNA virus belonging to the *Orthomyxoviridae* family. It infects humans and a wide range of animals, including wild birds, ducks, chickens, turkeys, pigs, horses, and bats [1], making it an important zoonotic pathogen responsible for global influenza pandemics and seasonal influenza outbreaks, thereby posing a serious threat to public health and the healthy development of the livestock industry. Despite the availability of vaccines and antiviral drugs, the high mutation rate of IAV frequently leads to mismatches between vaccine strains and circulating strains, as well as the emergence of drug-resistant variants. Influenza diversification occurs through two main mechanisms, including antigenic drift and antigenic shift [2,3]. Major pandemics, including the 1918 Spanish flu (H1N1), 1957 Asian flu (H2N2), 1968 Hong Kong flu (H3N2), and the 2009 pandemic (H1N1)pdm09, all arose from antigenic shifts [3,4]. Vaccine effectiveness has varied considerably, and the emergence of drug-resistant influenza strains further complicates treatment. M2 inhibitors like amantadine, once widely used since the 1960s [5], are no longer recommended due to widespread resistance-conferring mutations that emerged in A(H3N2) viruses between 2003 and 2006 and in A(H1N1) viruses in 2009. Moreover, resistance to neuraminidase inhibitors [6] and the polymerase acidic protein inhibitor baloxavir [7] has also been documented, though the prevalence of such resistance varies depending on the specific drug and the viral background strain. These challenges underscore an urgent need to develop novel antiviral strategies that can complement existing vaccines and therapeutics. Successful IAV replication relies heavily on the dual hijacking of host cellular metabolism and immune machinery, a process that drives lactate accumulation and subsequent lactylation. Therefore, a deep understanding of the molecular mechanisms governing these virus-host interactions is essential for identifying new therapeutic targets [8,9]. It should be noted that research on lactylation in IAV infection is still in its early stages, with only a limited number of direct studies currently available. Therefore, this review adopts a forward-looking, hypothesis-generating approach: we summarize the established mechanisms of lactylation in other viral systems and, based on these insights, propose testable hypotheses and key knowledge gaps for future IAV research.

Post-translational modifications (PTMs) represent a diverse array of covalent protein decorations that expand the functional complexity of the proteome beyond the limits of genetic encoding. By dynamically modulating protein activity, stability, subcellular localization, and interaction networks, PTMs govern nearly all aspects of cellular life. Within the intricate landscape of virus-host interactions, an expanding repertoire of PTMs, including palmitoylation, myristoylation, glycosylation, phosphorylation, acetylation, succinylation, ubiquitination, SUMOylation, NEDDylation, ISGylation, and lactylation, has been demonstrated to critically influence viral replication, assembly, egress, and immune evasion [10]. On one hand, viruses hijack the host PTM apparatus to promote their own life cycles; on the other, hosts deploy PTMs to neutralize viral protein functions [11]. In IAV infection specifically, phosphorylation, acetylation, and ubiquitination have been extensively characterized in the context of viral replication and immune evasion [12,13]. In 2019, Zhao and colleagues unveiled an entirely new modification, histone lysine lactylation (Kla), demonstrating that lactate can directly modulate gene transcription via covalent histone modification [14]. Using bacterially challenged M1 macrophages as an experimental system, they showed that histone lactylation functions as an epigenetic “lactate clock,” orchestrating homeostatic gene expression during the late phase of macrophage polarization. This breakthrough opened an entirely new avenue of metabolic-epigenetic crosstalk and has since catalyzed extensive research into the roles of lactylation in cancer, inflammation, and infectious diseases.

Lactylation is a covalent modification that attaches a lactyl group to lysine residues, a process fundamentally dependent on intracellular lactate levels and the availability of its activated precursor, lactyl-CoA. Viral infections can drive host metabolic reprogramming toward enhanced aerobic glycolysis, a phenomenon often referred to as the Warburg effect, which results in substantial lactate production [15]. In the case of IAV, multiple lines of evidence have shown that the virus stabilizes hypoxia-inducible factor 1α (HIF-1α) and elevates the expression of key glycolytic enzymes including HK2 and LDHA, leading to markedly increased lactate levels [16,17]. Beyond providing energy and biosynthetic building blocks for viral replication, this metabolic shift positions lactate as a signaling molecule that reshapes host immune responses through lactylation.

Recent studies have revealed that components of the IAV ribonucleoprotein (vRNP) complex undergo lactylation during infection, and this modification may facilitate vRNP-mediated transcription and replication [18]. Counteracting this process, the host delactylase SIRT1 exerts antiviral activity by interacting with vRNP components and catalyzing their delactylation, thereby suppressing vRNP function and limiting viral genome replication [18]. Notably, IAV infection leads to a marked reduction in SIRT1 expression. This downregulation not only maintains the lactylation status of viral vRNP proteins to support efficient replication but may also compromise SIRT1-mediated delactylation of host antiviral proteins such as IRF9.

Despite these insights, the comprehensive role of lactylation in IAV infection remains poorly understood. For example, it is unclear how IAV downregulates SIRT1, whether additional IAV-encoded proteins beyond vRNP components are subject to lactylation, whether key host innate immune proteins such as cGAS, MAVS, STING, or IRF3 undergo lactylation during IAV infection and what functional consequences ensue, and whether pharmacological targeting of the lactylation machinery could serve as a viable antiviral strategy against IAV. This review aims to synthesize current knowledge on lactylation in IAV infection, focusing on how IAV infection drives lactate accumulation and lactylation, how lactylation modulates host antiviral immune responses, and how IAV exploits lactylation to evade host immunity. Based on these insights, we will evaluate the therapeutic potential of targeting the lactylation machinery in IAV infection, discuss current limitations in the field, and propose future research directions to guide further investigation.

## IAV-induced metabolic reprogramming and lactate accumulation

### IAV infection enhances glycolysis

IAV infection drives a marked reprogramming of host glucose metabolism, with upregulated aerobic glycolysis standing out as a dominant and extensively documented feature. Infected lung epithelial cells display hallmarks of the Warburg effect, including heightened glucose uptake and elevated lactate output [19]. At the molecular level, viral proteins including NP and NS1 promote the stabilization and activation of HIF-1α, a central transcriptional regulator of glycolytic genes, thereby increasing the expression of key enzymes such as HK2, PFK, and LDHA [16,17]. Moreover, a recent investigation identified ARRDC4 as a novel sensor of IAV infection. ARRDC4 binds to the viral PA protein and enhances the enzymatic activity of muscle-type phosphofructokinase (PFKM) through interaction with its His298 residue, resulting in increased production of the glycolytic intermediate fructose-1,6-bisphosphate (FBP) [20].

The reliance of IAV on glycolysis for efficient replication has been probed using the glycolytic inhibitor 2-DG. In cultured cells, 2-DG treatment produces a dose-dependent reduction in IAV titers, an effect reversible by supplying exogenous glycolytic metabolites such as pyruvate or mannose, confirming that the observed inhibition stems specifically from glycolytic blockade [19]. At the mechanistic level, 2-DG prolongs viral mRNA synthesis while markedly diminishing the accumulation of genomic vRNA, indicating that glycolytic flux is essential for the viral polymerase to properly transition from transcription to replication [19,21]. Beyond glycolysis itself, other glucose-linked metabolic routes, including glutaminolysis, fatty acid synthesis (FAS), oxidative phosphorylation (OXPHOS), and the pentose phosphate pathway (PPP), also contribute to IAV replication, as disrupting them upsets the cellular glycolysis-respiration equilibrium and compromises vRNA production [21].

Although 2-DG reliably suppresses IAV replication *in vitro*, its effects in mice prove more nuanced. 2-DG administration has been shown to exacerbate disease indicators, hypoxemia, lung dysfunction, and humoral inflammation, without lowering viral loads [19]. Conversely, treatment with dichloroacetate (DCA), which directs pyruvate toward the TCA cycle, or rotenone (ROT), an OXPHOS inhibitor, alleviated IAV-driven pulmonary pathology without affecting viral titers [19]. These observations imply that the glycolytic shift triggered by IAV may actually confer host protection *in vivo*.

### Biological significance of lactate accumulation

Lactate accumulation during IAV infection serves multiple interconnected functions that span metabolism, pH regulation, and immune modulation.

#### Metabolic and biophysical support for viral replication

Lactate production is coupled with the regeneration of NAD^+^ and the generation of ATP and biosynthetic precursors, including nucleotides, amino acids, and lipids, all of which are essential for viral genome replication and progeny assembly. This metabolic strategy is shared across diverse viral systems. For example, foot-and-mouth disease virus (FMDV) increases HK2 expression and lactate accumulation to support replication [22], while classical swine fever virus (CSFV) induces L-lactate production via an LDHA-dependent axis that further modulates mitophagy and JAK-STAT signaling [23]. Beyond fueling biosynthesis, the acidic microenvironment created by lactate accumulation can promote IAV membrane fusion by inducing conformational changes in hemagglutinin (HA). Additionally, excessive lactate impairs mitochondrial function and disrupts cellular energy homeostasis. In dengue virus (DENV) infection, virus trigger ER-mitochondrial dysfunction that drives lactate overproduction, and inhibiting lactate production reduces viremia and mortality [24]. In HBV-expressing cells, lactate accumulation correlates with PDK activation, which blocks pyruvate-to-acetyl-CoA conversion, limits substrate availability for mitochondrial oxidative phosphorylation, and induces cellular stress [25].

#### Lactate as a signaling molecule and driver of protein lactylation

Beyond its classical metabolic roles, lactate functions as a signaling molecule that reprograms host immune responses via protein lactylation. In Singapore grouper iridovirus (SGIV) infection, lactate accumulation drives lactylation of key signaling proteins, including CaM, MEK1/2, ERK1/2, Hsp90, and Hsp70, indicating that lactylation is a central mechanism for host cell reprogramming during viral infection [26]. Functional experiments further showed that exogenous lactate suppresses SGIV replication, highlighting a context-dependent antiviral effect [26]. In human cytomegalovirus (HCMV) infection, lactate induces protein lactylation to promote viral spread, and dynamic lactylation of immune factors suppresses antiviral immunity. Specifically, K90 lactylation of IFI16 blocks DNA-PK recruitment, thereby preventing IFI16-driven gene repression and cytokine induction [27]. Importantly, lactate potentiates IFN-I at physiological or moderately elevated levels but exerts proviral effects at high concentrations, as demonstrated in the IRF9 lactylation study [28].

#### Lactate in disease pathogenesis

Lactate accumulation also contributes to tissue-specific pathogenesis. In enterovirus A71 (EV-A71)-induced viral muscle soreness, the HMGB1/LCN2/PDK1/lactate axis promotes lactate buildup in muscle and drives pain development [29]. In Epstein-Barr virus (EBV)-associated colorectal cancer, EBV-miR-BART18-3p activates the HIF-1α/LDHA axis by targeting SIRT1, leading to lactate accumulation and acetyl-CoA production, which in turn fuels de novo lipogenesis and tumor metastasis [30]. In HBV-related hepatocellular carcinoma (HCC), integrative lactylome analysis identified 9,275 Kla sites, with K28 lactylation of adenylate kinase 2 (AK2) inhibiting its activity and thereby promoting HCC proliferation and metastasis [31]. Collectively, these examples underscore that lactate accumulation is not merely a metabolic byproduct but an active driver of viral replication, immune evasion, and pathogenesis. However, in the specific context of IAV infection, direct evidence linking lactate to lactylation of specific host immune proteins remains limited, representing a critical knowledge gap.

### Lactylation landscape during IAV infection

IAV infection induces a global increase in protein lysine lactylation (Kla) levels, as evidenced by pan-Kla antibody staining in infected cells [18]. This observation aligns with the well-established concept that viral infections, including IAV, trigger metabolic reprogramming toward aerobic glycolysis, thereby providing lactate as the direct substrate for this modification [32]. Importantly, this lactylation signature is not restricted to IAV; it has been documented across a wide range of DNA and RNA viruses, often with distinct mechanistic outcomes.

#### Lactylation of viral proteins in IAV

Recent work has demonstrated that multiple components of the vRNP complex, specifically NP, PA, PB1, and PB2, undergo lactylation during infection. Functional evidence suggests that this modification contributes to vRNP transcriptional and replicative activity, positioning viral protein lactylation as a direct facilitator of the IAV life cycle [18].

#### Lactylation of host proteins: knowns and unknowns

**Figure 1.**
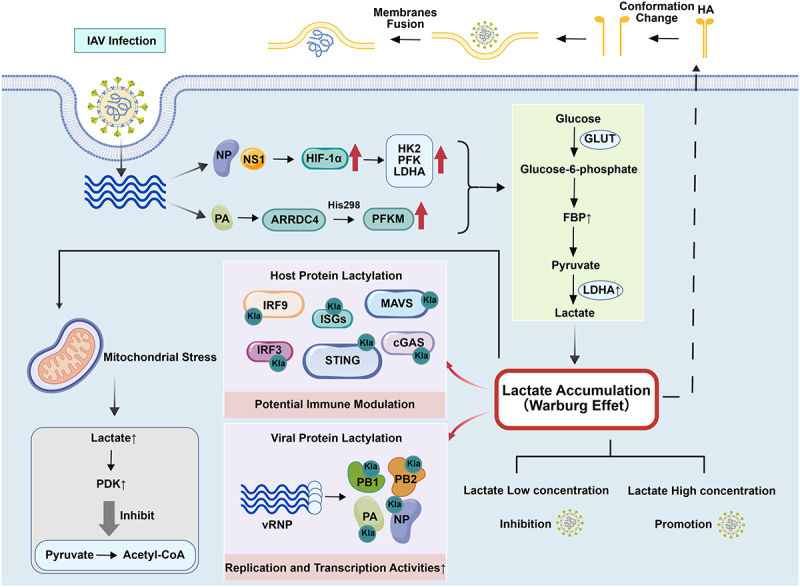
Schematic diagram of IAV-induced metabolic reprogramming and lactylation mechanism patterns. IAV infection reprograms host glucose metabolism via HIF-1α-mediated upregulation of HK2, PFK, and LDHA, while ARRDC4 enhances PFKM activity to augment glycolytic flux. The resulting lactate accumulation drives widespread lysine lactylation (Kla), including direct lactylation of vRNP components to promote viral replication, though the lactylation status of key host immune proteins (cGAS, MAVS, STING, IRF3) remains an open question. Lactate also activates PDK, inhibiting pyruvate-to-acetyl-CoA conversion and inducing mitochondrial stress, while promoting HA-mediated membrane fusion. Notably, physiological lactate levels suppress viral infection, whereas elevated levels promote viral propagation.

In contrast to viral protein lactylation, the lactylation landscape of host proteins during IAV infection remains incompletely characterized, as summarized in Figure 1. The reported lactylated proteins in IAV and other viral systems are also summarized in Table 1.

**Table 1.** Reported lactylated proteins during IAV infection and other viral systems.

Virus	Protein	Lactylation site	Biological function	Regulatory effect	Reference
IAV	NP, PA, PB1, PB2 (vRNP)	Not specified	Promotes vRNP transcriptional and replicative activity	Pro-viral	[18]
IAV	SIRT1	N/A	Removes lactyl groups from vRNP; suppresses viral replication	Antiviral	[18]
HBV	CENPA	K124	Transcriptional regulator cooperating with YY1; promotes HCC	Pro-viral	[33]
HBV	H3K56la (histone)	K56	Enhances OCT4 expression; promotes tumorigenesis of LCSCs	Pro-viral	[33]
HPV16	G6PD	K45	Promotes G6PD dimer formation; activates PPP; supports nucleotide synthesis	Pro-viral	[34]
KSHV	NAT10	K290	Promotes ac4C modification of tRNASer-CGA-1–1; facilitates viral reactivation	Pro-viral	[35]
KSHV	ALKBH5	Not specified	Promotes IFN-β mRNA biogenesis via m^6^A demethylation	Antiviral	[36]
SFTSV	YTHDF1	K517, K521	Promotes YTHDF1 degradation; counteracts host-mediated degradation of viral mRNA	Pro-viral	[37]
PRRSV	H3K18la (histone)	K18	Activates HSPA6 expression; impedes TRAF3-IKKε interaction; suppresses IFN-β	Pro-viral	[38]
WSSV	H3K18la, H4K12la	K18, K12	Upregulates S6K2 expression; promotes viral infection	Pro-viral	[39]
CSFV	H2BK16la (histone)	K16	Activates NF-κB pathway via KPNA2; induces IFN-λ expression	Antiviral	[40]
HCMV	IFI16	K90 (IDR)	Blocks DNA-PK recruitment; prevents IFI16-driven gene repression	Pro-viral	[27]

Several host proteins have been reported to undergo lactylation in other pathological contexts, including IRF9 and cGAS [28,41]. However, direct evidence for lactylation of these or other host proteins specifically during IAV infection is currently lacking. Whether critical innate immune proteins, such as cGAS, MAVS, STING, IRF3, or other interferon-stimulated genes, become lactylated in IAV-infected cells, and what functional consequences such modifications might have, represent important open questions. Addressing these gaps will be essential for a complete understanding of how lactylation shapes host antiviral immunity during IAV infection.

### Crosstalk between lactylation and other post-translational modifications

Emerging evidence indicates that lactylation does not function in isolation but rather engages in complex crosstalk with other PTMs, including acetylation, succinylation, ubiquitination, and RNA modifications. This crosstalk occurs at multiple levels: competition for shared lysine residues, common regulatory enzymes, and sequential modification cascades.

#### Lactylation and acetylation

Lactylation and acetylation share the same lysine residues on many proteins and are regulated by overlapping sets of “writer” and “eraser” enzymes. p300 functions as both an acetyltransferase and a lactyltransferase, while HDAC1-3 and SIRT1-3 remove both acetyl and lactyl groups [42,43]. This shared machinery creates direct competition between the two modifications. Notably, lactylation and acetylation can exert distinct functional outcomes on the same protein. For example, acetylation of cGAS at K384 promotes its activation, whereas lactylation at K21 promotes its degradation [44]. Understanding this competitive interplay is critical for interpreting PTM studies, as many early “acetylation” studies may have been confounded by the presence of lactylation [45].

#### Lactylation and succinylation

Succinylation, another acylation modification driven by succinyl-CoA, has also been implicated in viral infections. Both lactylation and succinylation are regulated by SIRT5 and SIRT7, though their metabolic origins differ [46–48]. Crosstalk between these modifications may coordinate metabolic adaptation during viral infection.

#### Lactylation and ubiquitination

Recent work has revealed a sequential crosstalk between lactylation and ubiquitination in antiviral signaling. Xie and colleagues demonstrated that TBK1 lactylation at K241, catalyzed by AARS1, activates IFN-I signaling [49]. Subsequently, SIRT6-mediated delactylation exposes the same lysine residue, allowing SIAH2 to catalyze K48-linked polyubiquitination and p62-mediated autophagic degradation of TBK1, thereby terminating the immune response [49]. This represents a cascade that fine-tunes antiviral immunity.

#### Lactylation and RNA modifications

Lactylation also crosstalks with RNA modifications such as m^6^A. ALKBH5, an m^6^A demethylase, undergoes lactylation during viral infection, which enhances its binding to IFN-β mRNA and promotes m^6^A demethylation, thereby increasing IFN-β mRNA stability and bolstering antiviral responses [36]. This represents a direct link between protein lactylation and epitranscriptomic regulation.

## Lactylation in innate antiviral immunity: lessons from other viral systems

This section summarizes established mechanisms of lactylation in other viral systems (e.g. HBV, HCMV, KSHV, PRRSV, SFTSV) to provide a reference framework for future IAV research. Direct IAV-specific evidence is highlighted where available.

### The cGAS-STING pathway

The cGAS-STING pathway is a central sentinel for cytosolic DNA, mediating IFN-I production in response to DNA viruses and, under certain conditions, RNA viruses. Upon recognition of double-stranded DNA (dsDNA), cGAS catalyzes the synthesis of the second messenger cGAMP, which activates STING and triggers downstream signaling cascades involving TBK1 and IRF3, ultimately inducing IFN-I and pro-inflammatory cytokines [50,51]. Although IAV is an RNA virus, infection can lead to mitochondrial damage and leakage of mitochondrial DNA (mtDNA) into the cytosol, thereby activating cGAS-STING signaling and contributing to the host antiviral response.

Accumulating evidence has established that cGAS lactylation serves as a potent negative regulatory mechanism, though its effects are highly context-dependent. The alanyl-tRNA synthetases AARS1 and AARS2 have been identified as intracellular L-lactate sensors that directly catalyze ATP-dependent lactylation on target proteins, including cGAS [41]. In response to L-lactate, AARS2 associates with cGAS and mediates its lactylation and inactivation. Using a genetic code expansion orthogonal system, the presence of a lactyl moiety at a specific cGAS amino-terminal site abolishes cGAS liquid-like phase separation and DNA sensing *in vitro* and *in vivo* [41]. Mechanistically, multiple studies have elucidated how cGAS lactylation leads to its functional inactivation. Lactylation of cGAS at K21 stimulates its translocation from the nucleus to the proteasome for ubiquitin-independent degradation, while lactylation at K415 rewires PIK3CB activity and impairs ULK1-driven phosphorylation of PSMA4 S188, further promoting cGAS degradation [44].

Notably, lactylation demonstrates significant heterogeneity in different pathological contexts. In systemic lupus erythematosus (SLE), cytosolic mtDNA stimulation triggers metabolic reprogramming in macrophages and dendritic cells, leading to a marked increase in lactate production. This metabolic byproduct specifically modifies cGAS through lactylation, altering its conformation, enhancing its stability, and preventing its interaction with the E3 ubiquitin ligase MARCHF5, thereby inhibiting ubiquitin-mediated proteasomal degradation. Stabilized cGAS continuously activates the STING signaling axis, amplifying the IFN-I response and forming a positive feedback loop of autoimmune inflammation [52]. Conversely, in sepsis-induced acute kidney injury, upstream cGAS-STING signaling promotes glycolytic reprogramming and lactate production, driving LDHB K156 lactylation and subsequent NLRP3 inflammasome activation [53]. In glioblastoma, inhibiting macrophage-derived lactate transport restores cGAS-STING signaling and enhances antitumor immunity [54]. In neuropathic pain, CMPK2 promotes microglial glycolysis and STING lactylation, leading to pathway deactivation [55], while in hypoxic-ischemic encephalopathy, lactate upregulates cGAS lactylation [56]. These context-dependent outcomes highlight the complexity of cGAS lactylation regulation, which can be either inhibitory or activating depending on the cellular and disease environment.

Despite the well-established role of cGAS lactylation in other diseases, direct evidence for this modification during IAV infection remains limited. However, several lines of indirect evidence suggest its potential relevance. First, IAV infection induces mtDNA release and cGAS-STING activation. Second, IAV infection promotes glycolysis and lactate accumulation, providing the substrate for cGAS lactylation. Third, MCT1 blockade has been shown to inhibit cGAS lactylation and restore innate immune surveillance against viral replication [41]. Key knowledge gaps include whether IAV-induced lactate accumulation drives cGAS lactylation in infected cells, which specific lysine residues are lactylated, and what the net effect on IAV replication and host outcomes might be. Addressing these questions will be essential for understanding the role of the cGAS-STING-lactylation axis in IAV pathogenesis.

### The RLR-MAVS pathway

The RIG-I-like receptor (RLR) pathway, particularly the RIG-I/MAVS axis, serves as the primary innate immune sensor for IAV RNA [57]. Upon recognition of viral RNA, RIG-I interacts with MAVS, triggering its aggregation and the subsequent activation of the TBK1-IRF3 axis to induce IFN-I production. A landmark study revealed that lactate functions as a natural suppressor of RLR signaling by directly binding to the transmembrane domain of MAVS, preventing its aggregation and thereby blocking signal transduction. This discovery established MAVS as a direct sensor of lactate, linking energy metabolism to innate immunity [58]. In addition, Thyrsted and colleagues provided direct evidence that lactate promotes IAV replication by inhibiting MAVS-dependent IFN-I production in primary human airway epithelium, demonstrating that IAV infection induces LDHA expression and LDHA-mediated lactate formation [59].

Notably, lactate-mediated MAVS suppression represents a conserved immune evasion strategy across multiple viral systems, though the underlying mechanisms exhibit context-dependent nuances. In hepatitis B virus (HBV) infection, the virus activates glycolysis and sequesters MAVS from RIG-I by forming a ternary complex that includes hexokinase (HK). HBV suppresses RLR signaling via LDHA-dependent lactate production, with lactate directly binding MAVS to prevent its aggregation and mitochondrial localization [60]. In porcine reproductive and respiratory syndrome virus (PRRSV) infection, the virus promotes glycolysis to produce lactate, which targets MAVS to inhibit RLR signaling and promote viral replication; glycolytic inhibitors targeting HK2 and LDHA effectively suppress PRRSV replication [61]. In Senecavirus A (SVA) infection, glycolysis induced by SVA facilitates virus replication by promoting lactate production, which attenuates the interaction between MAVS and RIG-I. SVA induces expression of HK2, PFKM, PKM, PGK1, HIF-1α, and SOD2, enhancing lactate production while reducing ATP generation [62].

Emerging evidence indicates that glycolysis and RLR signaling exhibit reciprocal inhibition, creating a regulatory feedback loop that shapes the outcome of viral infection. During RLR activation, glycolysis is inactivated, serving as a barrier to impede IFN-I production. RLR-triggered MAVS-RIG-I recognition hijacks hexokinase binding to MAVS, leading to impairment of its mitochondrial localization and activation. Lactate serves as the key metabolite responsible for glycolysis-mediated RLR signaling inhibition, and lactate restoration reverses the increased IFN production caused by lactate deficiency. Notably, lactate reduction via LDHA inactivation enhances IFN-I production and protects mice from viral infection [58,63]. The mammalian target of rapamycin (mTOR) positively regulates RLR-mediated antiviral activity in human dendritic cells, as RLR stimulation increases mTORC1 and mTORC2 phosphorylation, while mTOR inhibition impairs the RLR-triggered glycolytic switch and decreases TBK1 phosphorylation [64]. During pregnancy, RLR signaling is modulated by hormones and lactate, and its dysregulation can lead to complications such as preeclampsia and preterm birth [65]. These findings collectively establish that lactate-mediated MAVS suppression is a conserved mechanism across multiple viruses, including IAV, and that targeting lactate production may restore RLR-mediated antiviral immunity.

### The IFN signaling pathway

Beyond modulating IFN production, lactylation regulates IFN signal transduction at multiple levels, including direct modification of signal transducers, epigenetic regulation of IFN gene expression, and crosstalk with RNA modifications. A recent study demonstrated that L-lactic acid promotes IRF9 L-lactylation via the lactyltransferase AARS1 [28]. This modification enhances the IRF9-STAT2 interaction, thereby potentiating IFN-I signaling and boosting antiviral immune responses. Importantly, the same study revealed that viruses can achieve immune evasion by promoting SIRT1-mediated delactylation of IRF9. Furthermore, metformin promotes IRF9 L-lactylation by both accumulating lactic acid and disrupting virus-induced IRF9-SIRT1 interaction, highlighting a potential therapeutic application [28]. This finding has important implications for IAV infection, where lactate levels fluctuate over the course of infection.

Notably, lactylation demonstrates significant heterogeneity in regulating IFN responses across different pathological contexts. In the tumor immune microenvironment, lactate accumulation induced by intratumoral microbiota promotes lactylation at the K852 site of RIG-I, altering the conformation of its RNA recognition domain and blocking MAVS signaling complex assembly, thereby suppressing NF-κB and IRF3 signaling. Concurrently, lactylation of the NLRP3 inflammasome inhibits caspase-1 activation, reducing IL-1β and IL-18 secretion, driving macrophage M2 polarization, enhancing Treg immunosuppressive function, and inhibiting CD8^+^ T cell cytotoxicity [66]. The TLR signaling adapter BCAP activates the PI3K-AKT-mTOR pathway to drive glycolytic metabolism, generating lactate that acts as a substrate for histone modification, mediating specific enrichment at the promoter regions of repair-related genes and promoting the transition of macrophages from a pro-inflammatory M1 phenotype to a reparative M2 phenotype [67]. These findings underscore the bidirectional regulatory characteristics of lactylation in maintaining immune homeostasis and pathogen defense.

Histone lysine lactylation (Kla) has emerged as an epigenetic mechanism that directly regulates gene transcription during viral infection. In classical swine fever virus (CSFV) infection, researchers observed fluorescent Kla signals in all four histones (H2A, H2B, H3, and H4), with H2B being the most abundant, and H2B K16 was identified as a key lactylation site. CSFV infection increased global H2B Kla and H2BK16la levels in an LDHA-lactate axis-dependent manner. Mechanistically, H2BK16la and pan Kla activate the NF-κB pathway by mediating p65 nuclear translocation via karyopherin α2 (KPNA2), thereby inducing IFN-λ expression and inhibiting CSFV replication [40]. In PRRSV infection, virus-induced lactylation activates the expression of heat shock 70 kDa protein 6 (HSPA6), a negative regulator of IFN-β. Using CUT&Tag combined with RNA-seq, researchers found that PRRSV-induced lactylation upregulates HSPA6, which impedes the interaction between TRAF3 and IKKε, thereby hindering IFN-β production. This activated lactate-lactylation-HSPA6 axis promotes viral growth by impairing IFN-β induction [38].

Beyond histones and IRF9, lactylation directly modifies key signaling molecules to regulate IFN responses. STAT1, a critical transcription factor downstream of both type I and type II interferon receptors, undergoes lactylation at lysine 193 (K193) via AARS1. This modification inhibits STAT1 binding to JAK2 and its phosphorylation, thereby disrupting tumor responsiveness to IFN-γ signaling and leading to reduced expression of downstream chemokines including CXCL9, CXCL10, and CXCL11, ultimately facilitating immune escape [68]. Additionally, the RNA m^6^A demethylase ALKBH5 undergoes lactylation during viral infections, including herpes simplex virus type 1 (HSV-1), Kaposi’s sarcoma-associated herpesvirus (KSHV), and mpox virus (MPXV). Viral infections enhance ALKBH5 lactylation by increasing its interaction with acetyltransferase ESCO2 and decreasing its interaction with deacetyltransferase SIRT6. Lactylated ALKBH5 binds IFN-β mRNA, leading to demethylation of its m^6^A modifications and promoting IFN-β mRNA biogenesis, thereby strengthening antiviral innate immune responses [36].

**Figure 2.**
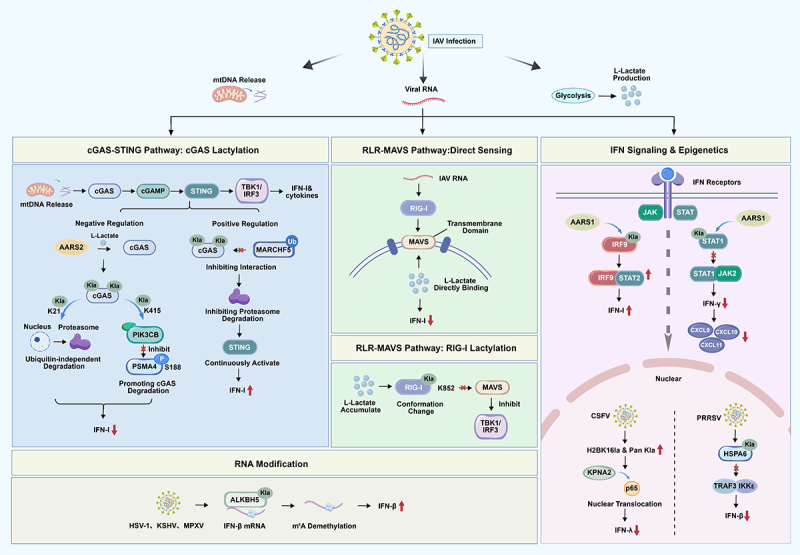
Lactylation regulates cGAS-STING, RLR-MAVS, and IFN signaling pathways. in the cGAS-STING axis, AARS1/2 catalyze cGAS lactylation (K21/K415), inactivating cGAS by suppressing phase separation or promoting degradation. In the RLR-MAVS pathway, lactate directly binds MAVS, blocking its oligomerization and inhibiting IFN production. RLR activation also reciprocally suppresses glycolysis. At the IFN signaling level, IRF9 lactylation enhances STAT2 interaction to boost IFN signaling, whereas STAT1 K193 lactylation suppresses IFN-γ responses. Histone lactylation activates NF-κB to induce IFN-λ, while PRRSV-induced lactylation upregulates HSPA6 to suppress IFN-β. Additionally, viral infection enhances ALKBH5 lactylation, promoting m^6^A demethylation of IFN-β mRNA and increasing its stability.

In the specific context of IAV infection, direct evidence for IRF9 lactylation, histone lactylation, or ALKBH5 lactylation remains limited. However, several lines of indirect evidence suggest their potential relevance. IAV infection induces SIRT1 downregulation, promotes glycolysis and lactate accumulation, and is known to dysregulate IFN signaling. It remains unknown whether histones are lactylated during IAV infection and whether such modification regulates IFN-related gene expression. Similarly, it has yet to be determined whether targeting IRF9 lactylation can enhance IFN signaling and suppress IAV replication. The regulatory network of lactylation in innate antiviral immunity is summarized in Figure 2.

## Lactylation in adaptive immunity: implications for IAV

Direct evidence for lactylation in T cells during IAV infection is currently lacking. This section draws on findings from tumor immunology and trained immunity studies to generate testable hypotheses for future IAV research.

### T cell responses and IAV clearance

T cell-mediated adaptive immunity is essential for the clearance of IAV and the establishment of protective immunological memory. CD8^+^ cytotoxic T lymphocytes (CTLs) eliminate virus-infected cells, while CD4^+^ helper T cells support B cell antibody production and CTL function. Upon activation, T cells undergo a profound metabolic shift from oxidative phosphorylation to glycolysis to meet the biosynthetic demands of proliferation and effector function [69]. Lactate is now recognized as a signaling molecule that shapes both adaptive and innate immune responses through histone modification and protein lactylation [70].

Histone lactylation has emerged as a key epigenetic mechanism linking T cell metabolism to function. In both human and mouse CD8^+^ T cells, enrichment of H3K18 lactylation (H3K18la) and H3K9 lactylation (H3K9la) has been observed, acting as transcription initiators of key genes regulating CD8^+^ T cell effector function. Distinct patterns of H3K18la and H3K9la are present in CD8^+^ T cell subsets, linked to their specific metabolic profiles. Modulation of these lactylation marks by targeting metabolic and epigenetic pathways influences CD8^+^ T cell effector function, including antitumor immunity, in preclinical models [69]. These findings suggest that similar mechanisms may operate during IAV infection, where CD8^+^ T cell activation and glycolysis are both enhanced.

Lactate exerts broad effects on CD4^+^ T cell subset differentiation. In the tumor microenvironment, lactate favors the conversion of naïve CD4^+^ T cells toward a regulatory phenotype while suppressing Th1 and Th17 lineages, thereby promoting immune suppression. This Th1 suppression occurs via SIRT1-mediated deacylation and subsequent degradation of T-bet, the lineage-defining transcription factor for Th1 cells [71]. Lactate also stimulates LDHA activity in naive CD4^+^ T cells, redirecting α-ketoglutarate toward 2-hydroxyglutarate production. This metabolic shift inhibits mTOR signaling, facilitating Treg differentiation and constraining the Th17 axis [72]. Furthermore, Th17 cells exposed to lactate undergo H3K18 lactation-mediated epigenetic reprogramming, transitioning toward a Treg-like phenotype characterized by reduced IL-17 production and increased Foxp3 expression in an IL-2-dependent manner [73]. Lactate also enhances Treg retention in inflamed tissues through MOESIN lactylation at residue K72, which strengthens its interaction with the TGF-β receptor I and amplifies SMAD3 signaling [74].

In the context of trained immunity, lactate release correlates with enhanced cytokine production upon re-stimulation. Trained monocytes and macrophages display H3K18la primarily at distal regulatory regions. This histone mark associates with active chromatin and persists after removal of the initial training stimulus, linking directly to the transcriptional program of trained immunity. Pharmacological blockade of lactate production or histone lactylation abrogates trained immunity responses, and genetic polymorphisms in LDHA and EP300 modulate this process. Notably, H3K18la persists for up to 90 days following BCG vaccination *in vivo*, establishing this mark as an epigenetic signature of innate immune memory [75,76].

### Memory T cell formation and IAV secondary infection

The transition from effector to memory T cells requires a metabolic shift from glycolysis-dominated metabolism back to oxidative phosphorylation. SIRT1 has been demonstrated to regulate memory T cell formation, as its deletion in human CD8^+^ T cells promotes effector differentiation while compromising memory development [77]. Given SIRT1’s additional function as a delactylase, lactylation may participate in the metabolic-epigenetic switch that controls memory T cell fate determination.

Supporting evidence from non-viral contexts includes studies on spinal cord injury, where glycolytic reprogramming in activated microglia and macrophages enhances histone lactylation-mediated Cxcl16 transcription, recruiting CXCR6^+^CD8^+^ T cells and exacerbating neuronal damage. Genetic or pharmacological inhibition of glycolysis ameliorates this effect [78]. In esophageal squamous cell carcinoma, elevated glycolysis drives lactate production, which induces H4K12la lactylation, upregulates PLAU expression, and dampens CD8^+^ T cell antitumor activity [79]. In small cell lung cancer, the LDHA-H4K5la-PRC2 axis suppresses antigen presentation, and LDHA inhibition sensitizes tumors to checkpoint blockade therapy [80]. Collectively, these findings illustrate that lactate-driven histone lactylation can profoundly influence CD8^+^ T cell function and antitumor immunity.

The effect of lactate on CD8^+^ T cells depends largely on pH conditions. The protonated form, lactic acid, dampens CD8^+^ T cell cytotoxic function by rerouting pyruvate metabolism away from pyruvate carboxylase and toward pyruvate dehydrogenase, thereby blocking succinate export and interfering with SUCNR1 activation [81]. Lactate also triggers PD-1 upregulation via GPR81 signaling, pushing CD8^+^ T cells toward an exhausted state [82], and reshapes the CD8^+^ T cell compartment by expanding effector memory and terminally differentiated effector memory subsets while elevating TOX expression, which in turn impairs perforin and granzyme B production [83]. By contrast, sodium lactate, which is less acidic, confers immune-protective benefits. Administration of sodium lactate raises intracellular lactate concentrations without changing extracellular pH, thereby strengthening CD8^+^ T cell stemness and antitumor immunity through H3K27 hyperacetylation-driven upregulation of Tcf7 [84]; early-activated CD8^+^ T cells are capable of taking up sodium lactate, which works together with HIF-1α to promote glycolytic metabolism and drive the expression of effector molecules such as IFN-γ [85]. Importantly, CD4^+^ and CD8^+^ T cells display divergent responses to lactate owing to their utilization of different lactate transporters. CD4^+^ T cells mainly rely on SLC5A12, a transporter that imports sodium lactate and sustains pro-inflammatory functions, whereas CD8^+^ T cells predominantly employ SLC16A1, which facilitates the entry of lactic acid and suppresses cytotoxic activity [86]. This distinction in transporter usage may hold important implications for understanding how lactate influences T cell responses during IAV infection.

### IAV-specific evidence and knowledge gaps

Direct evidence for lactylation-mediated regulation of T cell responses during IAV infection remains limited, though several observations suggest potential relevance. IAV infection elicits a robust CD8^+^ T cell response accompanied by glycolytic reprogramming and lactate production, and histone lactylation marks (H3K18la, H3K9la, H4K12la, H4K5la) regulate T cell effector function in other disease contexts, while trained immunity studies indicate that lactate production and histone lactylation establish innate immune memory that may inform IAV vaccination strategies. It remains to be determined whether IAV-induced lactate accumulation drives histone lactylation in virus-specific CD8^+^ T cells and modulates their effector function, and whether targeting lactate production or lactylation can enhance CD8^+^ T cell-mediated clearance of IAV.

## IAV exploitation of lactylation for immune evasion

### IAV downregulates SIRT1 to maintain viral protein lactylation

It has been demonstrated that IAV infection significantly downregulates SIRT1 expression [18]. SIRT1, an NAD^+^-dependent deacetylase and delactylase, functions as a key “eraser” of lysine lactylation marks on both histone and non-histone proteins [42,43]. Functional studies have demonstrated that SIRT1 depletion markedly enhances IAV replication, whereas SIRT1 overexpression suppresses it [18]. This downregulation appears to serve as a critical immune evasion strategy, conferring dual benefits to the virus. The primary benefit is the preservation of vRNP lactylation to sustain viral replication. SIRT1 interacts with IAV NP, PA, PB1, and PB2 proteins and restrains the activity of the vRNP complex, thereby repressing transcription and replication of the IAV genome. During IAV infection, viral NP, PA, PB1, and PB2 proteins undergo lactylation, and SIRT1 facilitates their delactylation, as evidenced by enhanced lactylation upon SIRT1 loss and impaired lactylation upon SIRT1 overexpression [18]. The second potential benefit involves modulation of host antiviral proteins. SIRT1 also acts as a delactylase for IRF9, and viruses can evade immunity by promoting SIRT1-mediated IRF9 delactylation [28]. In IAV infection, SIRT1 downregulation would be expected to increase lactylation of its substrates, including IRF9. However, because IRF9 lactylation enhances IFN-I signaling [28], increased IRF9 lactylation would benefit the host rather than the virus. This apparent paradox suggests that IAV may employ additional mechanisms to directly inhibit IRF9 lactylation, other delactylases may compensate, or the net effect may be cell-type or context-dependent. Notably, SIRT1 also regulates histone H3K18 lactylation, where SIRT1 overexpression decreases H3K18la while knockdown increases it, suggesting that SIRT1 downregulation during IAV infection may broadly impact the host epigenome [87,88].

Beyond its roles in lactylation, SIRT1 is a master regulator of cellular metabolism, inflammation, and stress responses. In cerebral ischemia-reperfusion injury, the SIRT1 agonist SRT1720 inhibits SOD2 lactylation, restores antioxidant activity, and suppresses ferroptosis [89]. In hyperuricemic nephropathy, lactylation homeostasis is restored via the SIRT1/p300 axis [90]. In heart failure, SIRT1 acts as a delactylase for α-MHC, and reduced SIRT1 activity worsens cardiac function [91]. In sepsis, lactate stimulates HMGB1 acetylation through Hippo/YAP-mediated SIRT1 suppression [92]. These observations indicate that SIRT1 downregulation during IAV infection may have broad consequences beyond vRNP delactylation, potentially affecting host antioxidant defenses, inflammatory responses, and tissue homeostasis.

### Lactylation of IAV viral proteins is required for efficient replication

Lactylation of viral proteins is functionally required for efficient viral replication. In IAV, multiple vRNP components undergo lactylation during infection, and SIRT1 interacts with these components to facilitate their delactylation. Consequently, loss of SIRT1 enhances vRNP lactylation and promotes viral replication, whereas SIRT1 overexpression has the opposite effect, and LDHA inhibition or depletion reduces vRNP lactylation, thereby abolishing the replication advantage conferred by SIRT1 deficiency [18].

This requirement for viral protein lactylation extends beyond IAV, suggesting a conserved mechanism across diverse viral systems. In sugarcane mosaic virus (SCMV) infection, LDH interacts with viral replication complexes (VRCs) and regulates their stability through lactylation, promoting viral replication [93]. In Singapore grouper iridovirus (SGIV) infection, lactyl-proteomic analysis reveals widespread lactylation on key viral and host proteins, and exogenous lactate at non-cytotoxic concentrations inhibits SGIV replication, indicating that proper lactylation levels are critical for viral fitness [26]. In human cytomegalovirus (HCMV) infection, lactate induces widespread protein lactylation to promote viral spread, with lactyllysine enriched in intrinsically disordered regions that regulate viral protein condensates and immune signaling [27]. In severe fever with thrombocytopenia syndrome virus (SFTSV) infection, the viral virulence factor NSs increases lactylation of YTHDF1 and promotes its degradation, thereby counteracting host-mediated degradation of m^6^A-marked viral mRNAs [37]. In HPV16 infection, the virus promotes G6PD dimer formation by inhibiting its lactylation at K45, activating the pentose phosphate pathway and promoting cell proliferation [34]. These examples illustrate that viruses can either promote or inhibit lactylation depending on their specific needs.

### The virus-host battle over lactylation/delactylation enzymes

Lactylation regulation involves a complex network of “writers” (lactyltransferases) and “erasers” (delactylases). On the writer side, the acetyltransferase p300 functions as a lactyltransferase capable of installing lysine lactylation marks on histones and other proteins [43]. More recently, the alanyl-tRNA synthetases AARS1 and AARS2 have been identified as intracellular L-lactate sensors and lactyltransferases that directly catalyze ATP-dependent lactylation on target proteins, including cGAS [41]. On the eraser side, systematic evaluation of zinc-dependent and NAD^+^-dependent histone deacetylases has identified HDAC1-3 and SIRT1-3 as delactylases capable of cleaving ε-N-L-lactyllysine marks, with HDAC1-3 showing robust activity toward both L- and D-lactyllysine as well as diverse short-chain acyl modifications [42,43]. SIRT1 and SIRT3 have been further characterized as key erasers with distinct substrate specificities toward histone and non-histone proteins [42].

In IAV infection, IAV significantly downregulates SIRT1 expression, an active viral strategy to restrain SIRT1-mediated delactylation of vRNP components and maintain their lactylation status [18]. However, the precise molecular mechanism by which IAV downregulates SIRT1 remains unknown, with possibilities including transcriptional repression, post-transcriptional mRNA destabilization, or protein degradation.

Beyond IAV, other viruses have evolved distinct strategies to manipulate the host lactylation machinery. SFTSV promotes lactylation of YTHDF1 to facilitate its degradation [37]; HCMV and HSV-1 induce dynamic lactylation of immune factors, including IFI16 K90 lactylation, to suppress immunity [27]; and HPV16 inhibits G6PD lactylation to activate the pentose phosphate pathway [34]. These examples demonstrate that viruses can either promote or inhibit lactylation depending on their specific needs.

Several key insights emerge from comparing IAV with other viruses. The targets of lactylation regulation vary widely, encompassing viral structural proteins (IAV vRNP components), viral replication complex components (SCMV VRCs), host restriction factors (SFTSV YTHDF1, HCMV IFI16), and host metabolic enzymes (HPV16 G6PD). The enzymatic machinery targeted also differs, with IAV targeting SIRT1 downregulation, HPV16 directly targeting G6PD lactylation, and SFTSV/HCMV promoting lactylation via unknown mechanisms. The evolutionary convergence of multiple viruses on lactylation as a regulatory node underscores the biological significance of this modification in virus-host interactions.

## Therapeutic targeting of lactylation in IAV infection

### Targeting lactate metabolism

Because lactate is the direct substrate for protein lactylation, interfering with lactate production or transport offers a rational antiviral strategy. Pharmacological agents that disrupt lactate metabolism have shown efficacy against multiple viruses, including IAV. LDHA catalyzes the conversion of pyruvate to lactate while regenerating NAD^+^, a critical step for sustaining glycolysis and ATP production. Inhibiting LDHA reduces intracellular lactate concentrations and consequently lowers global lactylation levels [94]. In IAV-infected cells, LDHA inhibition or depletion markedly reduces lactylation of the vRNP components, thereby eliminating the replication advantage conferred by SIRT1 deficiency [18].

Oxamate, a well-established LDHA inhibitor, suppresses glycolysis and viral replication in HBV, PRRSV, and SVA infections [60–62,95]. In WSSV-infected shrimp, LDH silencing significantly reduces viral loads and mortality [39]. In the context of influenza, MXSG treatment decreases expression of glycolysis-related enzymes, lowers lactate accumulation and HMGB1 lactylation, and reduces autophagosome accumulation. Docking analysis suggests that buddleoside, an active compound in MXSG, may interact with LDHA, pointing to natural products as potential LDHA-targeting antivirals [96]. Additionally, the LDHA inhibitor FX-11 has shown efficacy in reducing lactate production in various disease models, though its application in IAV infection requires further validation.

Beyond intracellular lactate metabolism, lactate transport also shapes the metabolic microenvironment. Monocarboxylate transporters MCT1 and MCT4 regulate lactate flux across the plasma membrane, with MCT1 facilitating import and MCT4 driving export [97]. Although MCT inhibitors remain largely unexplored in viral infections, cancer and immunology studies suggest their potential relevance. The MCT1/4 inhibitor AZD3965 reduces lactate export, reprograms metabolism, and promotes immune cell activity to inhibit tumor progression [98]. Notably, virus-specific MCT1-deficient CD8^+^ T cells fail to establish memory T cells, which are critical for controlling γ-herpesvirus reactivation, highlighting the importance of lactate export for protective memory T cell formation [99]. In cancer, the MCT1/4 inhibitor syrosingopine suppresses the growth of adult T-cell leukemia-lymphoma cells [100].

Targeting glycolysis upstream of lactate production offers another approach. The glycolysis inhibitor 2-DG reduces lactate production and viral load in PRRSV-infected cells, while the glycolysis activator PS48 has the opposite effect [101]. Demethylzeylasteral (DML), a triterpene compound, suppresses tumorigenesis by reducing lactate levels and inhibiting H3 histone lactylation [102]. MXSG, a traditional Chinese medicine formulation, reduces IAV NP levels and alleviates lung inflammatory injury *in vivo*, effects associated with decreased glycolytic enzyme expression, reduced lactate accumulation, and lower HMGB1 lactylation [96].

Despite these promising findings, several challenges remain. The concentration-dependent dual effects of lactate suggest that complete elimination may be detrimental, and systemic inhibition of lactate production could have off-target effects in highly glycolytic tissues such as the brain, retina, and testis. Moreover, *in vivo* outcomes are demonstrated by 2-DG worsening IAV disease in mice despite its antiviral effects in cell culture, highlighting the need for targeted delivery strategies.

### Targeting SIRT1

SIRT1, an NAD^+^-dependent deacetylase and delactylase, is a central regulator of antiviral immunity, inflammation, and cellular metabolism. Because IAV infection downregulates SIRT1 to maintain vRNP lactylation and promote viral replication [18], pharmacological activation of SIRT1 represents a logical antiviral strategy. SIRT1 activators, including resveratrol and the more potent synthetic compounds SRT1720 and SRT2104, have shown efficacy in various disease models. In COVID-19, SIRT1 activation suppresses NF-κB activity and reduces proinflammatory cytokine secretion, mitigating cytokine storms. SIRT1-driven autophagy also facilitates viral particle clearance and reduces viral replication [103].

However, the role of SIRT1 in viral infections is complex and context-dependent. In RSV infection, SIRT1 inhibition restores glucocorticoid sensitivity, but EX-527-mediated SIRT1 inhibition exacerbates lung pathology and increases viral load [104]. Dendritic cell-specific SIRT1 knockout exacerbates RSV-induced lung pathology, suggesting a protective role for SIRT1 through DC-mediated autophagy [105]. In LCMV infection, SIRT1 inhibitors enhance viral clearance, and age-associated miR-181a deficiency leads to SIRT1-mediated histone gene repression, causing replication stress in T cells; SIRT1 inhibition restores cell cycle progression and improves viral clearance in older individuals [106]. In HCV infection, the ΔNp63-miR-181a-SIRT1 pathway regulates T cell senescence, with SIRT1 overexpression impairing T cell responses [107]. In HBV infection, HBx induces USP26 to stabilize SIRT1, promoting HCC tumorigenesis [108], while HBsAg and HBeAg upregulate SIRT1 to promote M2 macrophage polarization via NICD deacetylation, contributing to immune evasion [109].

The dual role of SIRT1 is further illustrated by its effects on IFN signaling. SIRT1 physically associates with STAT1 and STAT3, deacetylating them at specific lysine residues, thereby downregulating their phosphorylation and inhibiting type I and type II IFN-induced signaling. SIRT1 knockout promotes ISG expression and suppresses viral replication, and the SIRT1 inhibitor EX527 produces similar effects [110]. Thus, SIRT1 can also function as a negative regulator of IFN signaling, potentially limiting antiviral responses. In the context of IAV, this complexity is particularly relevant. While SIRT1 activation would promote vRNP delactylation and suppress viral replication, it might also enhance STAT1/STAT3 deacetylation and inactivation, potentially blunting IFN signaling. The net effect of SIRT1 activation on IAV infection therefore requires careful evaluation.

### Challenges and future directions

Several pharmacological agents have been developed to target lactylation at different levels, including lactate metabolism, SIRT1 activity, and AARS1/2 lactyltransferases (Table 2). Despite these promising findings, several significant challenges must be addressed before clinical translation can be realized.

**Table 2.** Therapeutic strategies targeting lactylation in IAV infection.

Target	Agent	Mechanism	Current status	Reference
LDHA	Oxamate, FX-11	Reduces lactate production and lactylation	Preclinical; efficacy in IAV models requires validation	[111,112]
LDHA	MXSG (natural product)	Inhibits LDHA activity; reduces HMGB1 lactylation	Preclinical; shows anti-IAV activity	[96,113,114]
MCT1/4	AZD3965, syrosingopine	Blocks lactate export; reprograms metabolism	Preclinical in cancer; not yet tested in IAV	[115,116]
SIRT1	Resveratrol, SRT1720, SRT2104	Activates SIRT1 to promote vRNP delactylation	Preclinical; efficacy in IAV requires validation	[117–119]
AARS1/2	Not yet available	Blocks lactylation at the source	Under development; no clinical candidates	[41,120]
Glycolysis	2-DG	Inhibits HK2 and glycolysis	Preclinical; *in vivo* efficacy concerns	[16,19,121]

#### Current limitations

First, the concentration-dependent dual effects of lactate present a fundamental obstacle. Lactate exhibits antiviral effects at physiological and moderately elevated levels but proviral effects at high concentrations [28]. This non-linear relationship implies that therapeutic interventions must aim to fine-tune, rather than simply suppress, lactate levels. Second, marked discrepancies between *in vitro* and *in vivo* outcomes pose a significant translational hurdle. While 2-DG consistently suppresses IAV replication in cell culture, it exacerbates disease in mice without reducing viral loads [19]. This paradox likely reflects the complex interplay between antiviral effects on infected cells and systemic effects on immune cell function, tissue metabolism, and organ physiology. Third, the cell-type and context-dependent functions of key regulatory enzymes such as SIRT1 add another layer of complexity. SIRT1 activation may exert antiviral effects by promoting vRNP delactylation in infected epithelial cells, yet simultaneously suppress IFN signaling in immune cells by deacetylating STAT1 and STAT3 [110]. The net outcome of systemic SIRT1 activation remains unpredictable without a comprehensive understanding of cell-type-specific effects.

#### Future directions

Looking forward, several future directions warrant particular attention. The development of specific inhibitors targeting AARS1/2 may offer a more precise way to block lactylation at its source without affecting other NAD^+^ -dependent processes [41,120]. Such inhibitors could potentially be developed through structure-based drug design. In parallel, high-throughput screening for selective lactylation modulators could yield next-generation therapeutics with improved specificity and safety profiles. Furthermore, the identification of clinically applicable biomarkers of lactylation status would facilitate patient stratification and monitoring of therapeutic response. Given the conserved nature of lactylation mechanisms across viruses, successful strategies developed for IAV may be broadly applicable to other viral infections, including SARS-CoV-2 and RSV. In addition, combination regimens that simultaneously target multiple nodes of the lactylation network may produce synergistic effects with lower individual drug doses, thereby minimizing off-target toxicity.

In conclusion, while the path to clinical translation presents substantial challenges, the critical role of lactylation in IAV replication and immune evasion provides a strong rationale for continued investment in this area.

## Conclusions and future perspectives

Since the discovery of histone lysine lactylation in 2019, substantial progress has been made in understanding its role in IAV infection, establishing lactylation as a critical molecular link connecting IAV-induced metabolic reprogramming to antiviral immunity. IAV infection triggers a profound glycolytic shift via HIF-1α-mediated upregulation of HK2, PFK, and LDHA, leading to lactate accumulation that supports viral replication. Lactate serves as the direct substrate for protein lactylation, with IAV infection inducing global increases in lysine lactylation levels. Viral vRNP components undergo lactylation, a modification required for optimal vRNP activity. The host delactylase SIRT1 plays a central antiviral role by catalyzing vRNP delactylation, and IAV counteracts this defense by downregulating SIRT1 expression. Lactylation regulates multiple host innate immune pathways, including cGAS-STING, RLR-MAVS, and IFN signaling. Comparison with other viruses reveals that lactylation can be either pro-viral or anti-viral depending on context, with viruses having evolved diverse strategies to manipulate this modification for immune evasion. Despite these advances, numerous critical questions remain unresolved, including the molecular mechanism by which IAV downregulates SIRT1; the precise identification of lactylation sites on NP, PA, PB1, and PB2 and whether individual sites have distinct functional consequences; whether other IAV viral proteins also undergo lactylation; and the lactylation status of host innate immune proteins during IAV infection. Addressing these questions will require well-designed *in vivo* studies that account for the concentration-dependent dual effects of lactate and the discrepancies between cell culture and animal models. A deeper understanding of how lactylation integrates with other metabolic and epigenetic pathways could reveal new nodes for therapeutic intervention.

### Translational outlook

LDHA inhibitors (oxamate, FX-11), MCT inhibitors (AZD3965), and SIRT1 activators (SRT1720, SRT2104) show preclinical promise. Major hurdles, such as lactate’s biphasic effects, poor inhibitor specificity, and *in vitro-in vivo* discordance, must be overcome. Future directions include AARS1/2-specific inhibitors, biomarker discovery, and combination regimens. Despite these challenges, targeting lactylation is a rational and promising antiviral strategy.

## Data Availability

Data sharing is not applicable to this article as no new data were created or analyzed in this study.
